# Rapidly Progressive Disseminated Sporotrichosis as the First Presentation of HIV Infection in a Patient with a Very Low CD4 Cell Count

**DOI:** 10.1155/2017/4713140

**Published:** 2017-09-25

**Authors:** Isis Cristine Morávia Ribeiro de Oliveira-Esteves, Guilherme Almeida Rosa da Silva, Walter de Araujo Eyer-Silva, Rodrigo Panno Basílio-de-Oliveira, Luciana Ferreira de Araujo, Carlos José Martins, Rogério Neves-Motta, Marcelo Costa Velho Mendes de Azevedo, Dario José Hart Pontes Signorini, Jorge Francisco da Cunha Pinto, Lívia Machado Moura, Rafael Jacyntho Laterça, Diogo Raphael Garcia de Oliveira Pereira, Isabela Vieira do Lago, Fernando Raphael de Almeida Ferry

**Affiliations:** Hospital Universitário Gaffrée e Guinle, Centro de Ciências Biológicas e da Saúde, Universidade Federal do Estado do Rio de Janeiro (UNIRIO), Rua Mariz e Barros 775, Tijuca, 20270-004 Rio de Janeiro, RJ, Brazil

## Abstract

Sporotrichosis is a human and animal disease caused by species of the* Sporothrix schenckii* complex. It is classically acquired through traumatic inoculation of fungal elements. Most frequently, sporotrichosis presents as a fixed cutaneous or as a lymphocutaneous form. A much smaller number of cases occur as cutaneous disseminated and disseminated forms. These cases require immediate diagnosis and management to reduce morbidity and mortality. We present the case of a 34-year-old male patient in whom the first presentation of HIV infection was a rapidly progressive sporotrichosis with multiple cutaneous lesions, a high fungal burden in tissues, and pulmonary involvement. He had an extremely low CD4 cell count (06/mm^3^). Treatment with amphotericin B deoxycholate led to complete clinical resolution. Sporotrichosis remains a neglected opportunistic infection among HIV-infected patients in Rio de Janeiro state, Brazil, and awareness of this potentially fatal infection is of utmost importance if treatment is not to be delayed and if potentially devastating complications are to be avoided.

## 1. Introduction

Sporotrichosis, traditionally known as “rose-handlers' disease,” is a human and animal disease caused by dimorphic fungal species of the* Sporothrix schenckii* complex, such as* S. brasiliensis* and* S. schenckii* sensu stricto [1]. Sporotrichosis is classically acquired through traumatic inoculation into skin or mucosa of fungal elements while handling soil, plants, organic matter, and decaying vegetation contaminated with its mycelia or conidia. It most frequently presents as a fixed cutaneous or as a lymphocutaneous form, since infection may track along dermal lymphatics leading to a nodular lymphangitis. A much smaller number of cases occur as cutaneous disseminated sporotrichosis (without extrategumentary disease) and as disseminated sporotrichosis (with extracutaneous and/or multiorgan involvement), most notably in human immunodeficiency virus- (HIV-) infected subjects [1]. These cases require immediate diagnosis and management to reduce morbidity and mortality.

Although sporotrichosis has been reported throughout the world, endemic areas are usually considered to be Latin America, South Africa, India, and Japan [2]. Sporotrichosis may occur in isolated cases, usually linked to certain occupational and leisure activities, such as gardening and hunting. It may also occur in outbreaks associated with certain occupations, such as forestry workers of reforestation programs [3], handling of mulching hay [4], and mine working [5]. In the last two decades, an unprecedented epidemic of sporotrichosis linked to zoonotic transmission of* S. brasiliensis* from scratches, bites, or simply contact with diseased cats has been reported in Brazil, mainly in Rio de Janeiro state [6, 7].

We wish to report the case of a 34-year-old male patient in whom the first presentation of the acquired immunodeficiency syndrome was a rapidly progressive sporotrichosis with multiple cutaneous lesions, a high fungal burden in tissues, and probable pulmonary involvement.

## 2. Case Report

A 34-year-old previously healthy male patient was referred to our university hospital with a two-month history of multiple cutaneous lesions. The first lesions were noted on the torso, but similar lesions rapidly followed on the arms, legs, and face. Lesions were not painful or pruritic, but some soon assumed an ulcerated and crusted aspect. As his illness progressed, he lost weight and a nonproductive cough developed. He also complained of night sweats but did not recall having had fever.

The patient was born and resided in the city of Rio de Janeiro and worked with cleaning and disinfection of water storage tanks and pipes. As part of his activities, he was frequently exposed to indoor and outdoor organic material, including bird droppings. He was a long-time smoker and gave a history of alcohol abuse and inhaled cocaine use. Topical and oral antimicrobial agents, such as azithromycin and cefuroxime, had been prescribed at another facility, with no clinical response.

**Figure 1:**
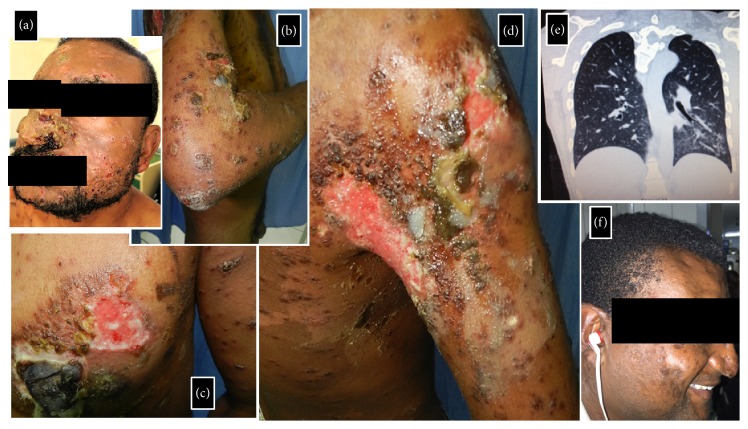
Clinical images of our 34-year-old male patient who presented with a 2-month history of rapidly spreading multiple cutaneous lesions. Annular brownish papules and plaques, with or without a scale crust, scattered over the face (a), chest (c/d), and extremities (b/d). Some larger lesions assumed a rupioid aspect (c). Large reddish shallow ulcers in right mammary (c) and right anterior axillary (d) regions. Some lesions drained a seropurulent discharge. Ground grass opacities are seen over the superior segment of the left inferior lobe (e). Complete remission of cutaneous lesions after treatment (f).

Clinical examination revealed a febrile and moderately undernourished patient with multiple brownish papules and plaques scattered over the trunk, face, and extremities (Figure 1). An annular format, with or without a scale crust, was typical of most early lesions. Some larger lesions, such as those in the* ala nasi*, were covered with a yellowish verrucous crust that could assume a rupioid aspect. In the right mammary and right anterior axillary regions there were large reddish shallow ulcers that reached several centimeters. Some lesions drained a seropurulent discharge (Figure 1).

Laboratory evaluations were remarkable for a normochromic, normocytic anaemia with a hemoglobin of 7.4 g/dl (13.8–17.2 g/dl), and reactive serologic tests for HIV infection, with a CD4 cell count of 06 cells/mm^3^. The patient was unaware of his HIV status. Serologic tests for hepatitis B, hepatitis C, and syphilis were negative. Three sputum samples were acid-fast bacilli negative. Blood and sputum samples yielded negative results on bacterial and fungal cultures.

**Figure 2:**
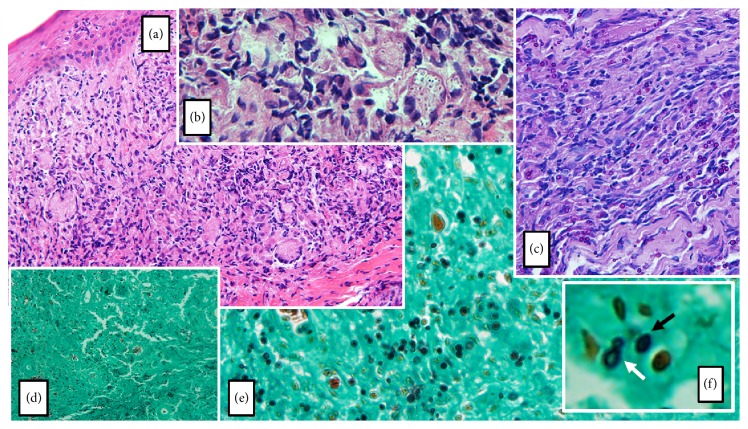
(a) Hematoxylin and eosin stain (original magnification 100x) discloses a dermal chronic granulomatous inflammatory reaction, with the presence of Langhans giant cells, epithelioid histiocytes, plasmocytes, neutrophils, and eosinophils. (b) Hematoxylin and eosin stain (original magnification 400x) shows abundant rounded structures located inside multinucleated giant cells and histiocytes. (c) Periodic acid-Schiff stain (original magnification 200x) discloses spherical fungal elements. (d) and (e) Grocott's methenamine silver stain (original magnification 100x and 200x, resp.) unmasks countless 2 to 6 *μ*m darkly stained round to oval, as well as some cigar-shaped, elongated unicellular yeast-like structures, consistent with* Sporothrix* spp. (f) A cigar-shaped structure (black arrow) and a narrow-based budding yeast (white arrow) are shown in detail.

Chest radiography and computed tomography revealed small nodular and confluent ground grass opacities over the posterior segment of the left upper lobe and superior segment of the left inferior lobe (Figure 1). Histopathological examination of skin biopsy samples yielded a chronic granulomatous inflammatory reaction, with the presence of Langhans giant cells and countless yeast-like structures, consistent with* Sporothrix* spp. (Figure 2). Skin biopsy samples were also sent for fungal cultures. These resulted positive for* Sporothrix* spp. Therefore, a diagnosis of sporotrichosis with multiple cutaneous lesions and probable pulmonary sporotrichosis was made. Molecular studies for species identification were not performed due to unavailability. On further history taking, the patient informed that he had never worked as a gardener or recalled having been stabbed with thorns but did have long-lasting contact with her neighbor's cats.

Treatment was initiated with a daily regimen of amphotericin B deoxycholate, starting with escalating doses. Trimethoprim-sulfamethoxazole and azithromycin were also prescribed as prophylaxis against opportunistic infections. After three weeks, when a cumulative dose of 0,7 g was reached, oral itraconazole 200 mg/d was substituted for amphotericin B deoxycholate. Highly active antiretroviral therapy was initiated, and no evidence of an immune reconstitution inflammatory syndrome was recorded. The patient slowly improved and was discharged two months after admission. There was no evidence of relapse after three years of follow-up.

## 3. Discussion

In recent years there has been an increased recognition of the burden of disseminated sporotrichosis among HIV-infected subjects. However, there is a scarcity of data on the association between HIV and sporotrichosis [8]. A recent systematic review highlighted the features of HIV-sporotrichosis interaction among 58 patients, 33 (56,9%) of whom from Brazil [8]. Patients were predominantly male (84,5%) with a mean CD4 cell count of 97 cells/mm^3^. A relatively low CD4 cell count was strongly associated with disseminated and cutaneous disseminated forms of the disease. The majority of cases reported from Brazil were due to zoonotic transmission by infected cats and, less often, dogs. The management of sporotrichosis in an HIV-infected patient may prove to be exceedingly difficult [9–11].

The varied clinical presentation patterns of sporotrichosis may be driven by factors such as the number and size of the initial inoculum, the immune status of the host, the depth of traumatic inoculation, resistance to treatment, and other factors related to differences in strain virulence. Our patient first presented with an extremely low CD4 cell count of 06/mm^3^. Histopathological examination revealed a very high fungal load in tissues, which is unusual in human sporotrichosis. A large histopathologic study of 119 samples of confirmed cases of cutaneous sporotrichosis in Rio de Janeiro reported that no fungal structures were seen in 77 (64.7%) of cases, whereas another 16 (13.4%) cases had a very low fungal burden [12]. A high fungal burden, such as that seen in our patient's biopsies, was found in only 5.9% of the cases [12]. In fact, in endemic areas of sporotrichosis, the diagnosis is highly suspected when the architectural pattern of deep mycosis is present in a section of tissue where no microorganism can be found [13]. It seems evident that our patient's severely compromised immunity played a role in the clinical and histopathological presentation.

It is not clear whether multiple cutaneous sporotrichosis lesions are a result of repeated inoculations or are due to hematogenous dissemination or even to self-inoculation. A study of 24 cases of culture-proven cat-transmitted sporotrichosis, who presented with widespread cutaneous lesions and were apparently unrelated to HIV, suggested that repeated inoculation was probably responsible for the clinical presentation in most cases [14]. Our patient's occupation probably offered multiple opportunities for environmental exposure to pathogenic* Sporothrix *spp. Moreover, he gave a history of long-lasting contact with his neighbor's cats. We are not aware whether some of these cats might had feline sporotrichosis.

Our patient also presented with pulmonary disease that was highly likely due to sporotrichosis, even though a microbiological diagnosis was not possible. Pulmonary sporotrichosis has rarely been reported in HIV-negative subjects [15]. Some of these patients had no cutaneous disease [16–18], raising the hypothesis that lung colonization occurs through inhalation of conidia. Lung disease seems to occur more frequently among HIV-infected patients, even though a microbiological diagnosis is achieved in only a small fraction of cases [8]. Histoplasmosis, a highly underdiagnosed fungal disease in Latin America [19], would stand as a major differential diagnosis. However, we strongly believe that the overall picture is consistent with pulmonary sporotrichosis.

We found this case to be noteworthy due to the aggressive clinical evolution in a span of two months, as the first complication of HIV infection. The histopathological demonstration of dermal tissues teeming with fungal structures highlights the potential aggressiveness of the fungal disease in the present case. Fortunately, prompt and complete response to appropriate treatment was achieved. Sporotrichosis remains a neglected opportunistic infection among HIV-infected patients in Rio de Janeiro state and awareness of this potentially fatal infection is of utmost importance if treatment is not to be delayed and if potentially devastating complications are to be avoided. The present case also seems to underline the importance of a having a heightened awareness of the overall burden of neglected fungal diseases in Brazilian patients [20].
